# Relationship of muscle function to circulating myostatin, follistatin and GDF11 in older women and men

**DOI:** 10.1186/s12877-018-0888-y

**Published:** 2018-08-30

**Authors:** Elizaveta Fife, Joanna Kostka, Łukasz Kroc, Agnieszka Guligowska, Małgorzata Pigłowska, Bartłomiej Sołtysik, Agnieszka Kaufman-Szymczyk, Krystyna Fabianowska-Majewska, Tomasz Kostka

**Affiliations:** 1 Department of Geriatrics, Healthy Ageing Research Centre, Medical University of Lodz, ul. Pieniny 30, 91-647 Łódź, Poland; 2 Department of Physical Medicine, Medical University of Lodz, Hallera 1, Łódź, Poland; 3 Department of Biomedical Chemistry, Medical University of Lodz, Mazowiecka 6/8, Łódź, Poland

**Keywords:** Functional performance, Sarcopenia, Quadriceps muscle power, Optimal shortening velocity, Aging

## Abstract

**Background:**

Myostatin, its inhibitor follistatin, and growth/differentiation factor 11 (GDF11) have been proposed as factors that could potentially modify biological aging. The study aimed to test whether there is a relationship between these plasma circulating proteins and muscle strength, power and optimal shortening velocity (υ_opt_) of older adults.

**Methods:**

The cross-sectional study included 56 women and 45 men aged 60 years and older. Every participant underwent examination which included anthropometric and bioimpedance analysis measurements, functional and cognitive performance tests, muscle strength of upper and lower extremities, muscle power testing with two different methods and blood analyses.

**Results:**

Women had higher plasma levels of myostatin and GDF11 than men. Men had higher plasma level of follistatin than women. In women, plasma level of myostatin was negatively correlated with left handgrip strength and υ_opt_. Follistatin was negatively correlated with maximum power output (P_max_), power relative to kg of body mass (P_max_∙kg^− 1^) (friction-loaded cycle ergometer) and power at 70% of the 1-repetition maximum (1RM) strength value (P70%) of leg press (Keiser pneumatic resistance training equipment), and positively correlated with the Timed Up & Go (TUG) test. GDF11 was negatively correlated with body mass, body mass index, waist circumference, fat mass and the percentage of body fat. In men, there were no significant correlations observed between circulating plasma proteins and muscle function measures.

**Conclusions:**

The circulating plasma myostatin and follistatin are negatively associated with muscle function in older women. There is stronger relationship between these proteins and muscle power than muscle strength. GDF11 has a higher association with the body mass and composition than muscle function in older women.

## Background

Population aging is transforming the world significantly. It is undeniable that the patient’s age is a primary risk factor for sarcopenia, frailty and disability. In recent years, there are various attempts by different research groups to figure out the nature of that link. Those efforts included the search for the circulating blood factors that could be identified as biomarkers of aging [1]. Myostatin, its inhibitor follistatin, and growth/differentiation factor 11 (GDF11) have been proposed as such factors that could potentially modify biological age [2, 3].

Myostatin is a strong negative regulator of skeletal muscle development and size [4]. It is a member of the transforming growth factor-β (TGFβ) family, acting through the activin type II A and B receptors. It is highly expressed in skeletal muscle, but also present in adipose tissue and cardiac muscle [5]. Follistatin is a glycosylated plasma protein, a member of the TGFβ family [6]. It is abundant in different tissues such as placenta, ovary, testis and skeletal muscles. It inhibits myostatin from binding to the activin type II B; therefore, neutralizing it in circulation [7]. GDF11 is strongly related to myostatin, also a member of the TGFβ family [8]. It is expressed in the pancreas, intestine, kidney, skeletal muscle, and nervous system [9]. In recent years, GDF11 has been described as a circulating age-associated factor with different proposed roles [1].

The changes in the body composition that occur with aging can negatively affect daily functioning and health status of older people. Loss of strength and skeletal muscle mass have been identified as prime risk factors for falls and impaired mobility in older people [10]. Falls are frequent reason for the emergency department visits of older adults [11]. Skeletal muscle strength is identified as the maximum capacity to develop force [12]. It is associated with activities of daily living and mobility [13]. Muscle power is defined as the ability to exert force as fast as possible [14]. Muscle power and contraction velocity are strong independent predictors of functional performance such as gait speed, chair-rise time, and stair-climb time in older adults [15–17]. Muscle power is also associated with dynamic balance and postural sway. It is thought to be a better predictor of fall risk than muscle strength [18].

In available literature, there are no records of studies that would consider all three mentioned above proteins and their relationship to those measures of muscle function. Therefore, the main aim of the study was to test whether there is a relationship between circulating myostatin, follistatin and GDF11, with muscle strength, power and optimal shortening velocity (υ_opt_) of older adults.

## Methods

### Participants

One-hundred and six subjects were consecutively recruited between October 2015 and May 2016 for the study through the local newspapers’ advertisements. To be eligible, subjects needed to be 60 years and older, community dwelling, able to understand and execute commands, and willing to participate and give blood samples. Exclusion criteria included: acute illness, unstable cardiovascular or metabolic disease, myocardial infarction in the past 6 months, upper or lower limb amputation, neuromuscular and musculoskeletal disorders disrupting the voluntary movements, and cognitive impairment. Out of those 106, 5 subjects were excluded due to the contraindications to muscle power testing. Therefore, 101 participants, all of them Caucasian, were finally included to the study. The average number of chronic illnesses was 2.9 ± 1.9. Fifteen participants (14.9%) were diagnosed with coronary heart disease, 13 (12.9%) had chronic heart failure, seven (6.9%) suffered from stroke, 10 (9.9%) had chronic pulmonary disease, 19 (18.8%) had gastrointestinal disease, and 8 (7.9%) had history of cancer. Fifty-three participants (52%) were treated for hypertension, 55 (54%) for hypercholesterolemia, and 19 (18.8%) for diabetes mellitus type 2. Osteoarthritis was diagnosed in 36 subjects (35.6%) and 19 (18.8%) had osteoporosis. Each participant signed written informed consent, which was an obligatory requirement for the study participation. The Medical University of Lodz Ethics Committee approved this study.

### Protocol

The examination lasted approximately 4 h per patient. It was performed in the Geriatric Outpatient Clinic, Central Veterans’ Hospital in Lodz (Poland). The participants reported to the Clinic after 8 h of overnight fasting for blood sampling. Then the interview on socioeconomic status, current and previous illnesses and current medications was conducted. The contradictions for bioimpedance testing (implanted cardiac device, high fever) were identified for each participant. All participants underwent medical examination, which included blood pressure measurement to identify possible contraindications to the muscle power testing. Thereafter, participants could eat breakfast (all subjects were instructed to bring sandwiches, beverages, and comfortable sportswear to the appointment). The anthropometric, skinfolds and muscle strength measurements were intermitted by functional and cognitive performance tests. The same researcher/research assistant was always responsible for conducting the measurements to avoid interobserver/−analyzer variability. Before the muscle power assessment, all the subjects underwent a 5-min warm-up on the friction-loaded cycle ergometer.

### Anthropometric measurements

The anthropometric measurements including height and body mass were obtained. Each patient was weighed on calibrated SECA chair scales without shoes and outdoor clothes to the nearest 0.1 kg. Height was measured with the stadiometer to the nearest 0.5 cm. Body mass index (BMI) was calculated as body mass in kilograms divided by the height in meters squared. Calf circumference (CC) was measured on the widest part of the left leg to the nearest centimeter without compressing the subcutaneous tissue with flexible plastic tape. Waist circumference (WC) was measured midway between the lower rib cage and the iliac crest to the nearest centimeter with flexible plastic tape. Hips circumference (HC) was measured on the widest part of the buttocks. Skinfolds measurements were obtained using Baseline Skinfold Caliper at four sites: triceps, biceps, subscapular and supraileum. The percentage of body fat was estimated from the skinfolds measurements, according to the Durnin and Womersley method [19].

### Body composition

The body composition of 93 study participants due to preexisting contradictions was also analyzed by validated electrical bioimpedance device (Maltron Bioscan 920, Maltron International Ltd., Rayleigh, Essex UK). Two injector electrodes were placed on the dorsal surface of the foot and wrist. Two detector electrodes were placed between the styloid process of radius and ulna, and between the medial and lateral malleolus. During the measurement, each subject remained in the supine position with feet apart and hands at their sides.

### Functional performance

Functional performance for each subject was assessed by several tests and scales: Activities of Daily Living (ADL) scale [20], Instrumental Activities of Daily Living (IADL) scale [21], Timed Up & Go (TUG) test [22], and Tinetti test [23]. ADL consists of 6 questions concerning basic daily activities: self-feeding, dressing, bathing, toileting, continence, and transferring. It is graded from 0 to 6 points. A poor score reflects the necessity of supervision by caregiver in performing basic daily activities by a patient. IADL scale includes 8 areas of instrumental activities of daily living: ability to use telephone, shopping, food preparation, housekeeping, mode of transportation, responsibility for own medication, ability to handle finances and laundry. It is graded from 0 (low function, dependent) to 8 (high function, independent) points. The TUG test assesses mobility: standing up from a chair, walking a 3-m distance, returning, and sitting down as quickly as possible. The test was timed using stopwatch to the nearest 0.1 s. The Tinetti test includes the assessments of balance and gait with the maximum of 28 points.

### Cognitive assessment

Global cognitive function was assessed by the Folstein Mini-Mental State Examination (MMSE) with scores ranging from 0 to 30 points [24]. The Geriatric Depression Scale (GDS) Short Form is a questionnaire consisting of 15 yes/no questions, which assesses depression in the previous week. A score greater than 5 indicates the increasing likelihood and severity of depression in the examined patient [25].

### Muscle strength

Muscle strength was measured with two different methods. Muscle strength of upper extremities was assessed by a handgrip test (evaluating flexors of the hand and forearm) using Jamar Hydraulic Hand Dynamometer (Lafayette Instrument, Lafayette, IN, USA). The handgrip test was performed in duplicate for both hands with best result recorded. The participant was in the standing position with arms along the side not touching the body. Participants were asked to squeeze the dynamometer with as much force as possible with a 20–30s pause between trials to avoid muscle fatigue. All subjects were given verbal encouragement during the trials to ensure full activation and generation of maximal muscle strength. The results of each trial were recorded to the nearest kilogram.

Muscle strength of lower extremities was quantitatively determined by the one repetition maximum (1RM) measure of bilateral leg extension (evaluating quadriceps) and leg press (evaluating quadriceps, hamstrings, gluteals) using Keiser pneumatic resistance training equipment fitted with A300 electronics (Keiser Sports Health Equipment, Fresno, CA). The 1RM is described as the maximum load than can be lifted once throughout the full range of motion while sustaining the correct technique [26]. For the leg extension, the subjects were asked to sit in the upright position and on the cue to extend right knee as fast and forceful as possible. The subjects were asked to cross their upper extremities on the chest, and to not lift the gluteals off the seat while performing the movement. The same procedure was repeated with the left leg. For the leg press, the starting position of the seat was adjusted to where the knee joint is as a 90°-degree angle between thigh and shin. The subjects were instructed to cross their upper extremities on the chest, and to extend fully both legs simultaneously but without locking the knee joints. The examiner progressively increased the resistance between 5 to 10 kg for each repetition until the subject could no longer move the lever arm for leg extension or pedals for leg press through the full range of motion. Each trial was separated by the 30-s rest period.

### Muscle power

Muscle power was measured with two different methods: using Keiser pneumatic resistance training equipment fitted with A300 electronics (Keiser Sports Health Equipment, Fresno, CA) and friction-loaded cycle ergometer. First method included measurements of leg extension and leg press at 40 and 70% of the 1RM. These two percentages of the 1RM were chosen to represent muscle power production of low force/high velocity (40%) and high force/low velocity (70%) [13]. Muscle power at 40 and 70% is related to functional performance such as stair-climb time, chair-rise time, and habitual gait speed. Muscle power at 40% 1RM is suggested to be a better predictor of walking performance [26]. Those assessments were performed with five repetitions using the same Keiser pneumatic resistance training equipment. The subjects were instructed to complete the movement of each repetition as fast as possible, then to slowly lower the weight. All the attempts were verbally cued. All subjects were highly motivated during the exercise testing to enhance their performance. Each trial was separated by the 30-s rest period. For the leg extension, the maximum values for each side were recorded for further analyses. For the leg press, the highest measured simultaneously for both legs values were recorded for further analyses.

The second method included measurement of muscle power using the friction-loaded cycle ergometer (Monark type 818E Stockholm, Sweden) [17, 27]. The ergometer was instrumented with a strain gauge (KMM20 type, 200 N, WObit, Poznań, Poland) and an incremental encoder (Rotapuls 141-H-200ZCU46L2 type, 200pts/turn, Lika Electronic, Carre, Italy) for measurement of the friction force applied by the tension of the belt that surrounded the flywheel and the flywheel displacement, respectively. Instantaneous pedaling velocity (υ), force (F), and the power output (P) were calculated each 5 ms and then averaged over each downstroke period. The saddle height of ergometer was adjusted to the maximum comfort of each subject. The subjects were familiarized with the ergometer by the 5-min of submaximal cycling and sprints of 3–4 s against different friction loads. Following the warm-up and 5-min rest, the subjects were instructed to perform 8 s sprints from a standardized starting position, each separated by at least 5-min break. Friction loads were 0.25 N·kg^− 1^ and 0.35 N·kg^− 1^ of body mass. The υ-P combinations obtained during two sprints were fitted by a least square mathematical procedure to establish the υ-P relationship. The highest value of P (maximum power output - P_max_) and optimal shortening velocity (velocity at which the power reaches a maximum value - υ_opt_) were calculated from a third-order polynomial function. P_max_ was also expressed as relative to the body mass: P_max_·kg^− 1^(W·kg^− 1^). υ_opt_ was given in the number of rotations per minute (rpm) [17].

### Laboratory analysis

#### Plasma samples

The venous 5 ml blood samples were drawn after overnight fasting into tubes containing EDTA. Samples were centrifuged at 3500 rpm for 20 min at 4 °C in the Eppendorf 5430R centrifuge (Eppendorf AG, Hamburg, Germany) and divided into aliquots. The isolated plasma was stored at − 80 °C until analyzed.

#### Plasma analysis

Plasma was analyzed using immunoassays, which utilize the quantitative sandwich enzyme immunoassay technique. A monoclonal antibody specific to the protein was pre-coated onto a microplate. The samples and standards were pipetted into the wells and any of the proteins of interest present could bind by the immobilized antibody. After washing away any unbound substances, an enzyme-linked monoclonal antibody specific to the proteins of interest is added to the wells. Following the consequent wash to remove the unbound antibody-protein complexes, the substrate solution was added to the wells and developed color was proportioned to the amount of proteins bound. The intensity of color was measured using absorption photometry technique.

Plasma myostatin levels were measured using 4-h immunoassay kits (Immundiagnostik AG, Bensheim, Germany) according to the included protocol by manufacturer. Samples were diluted 1:10 prior to assay. Samples were analyzed in duplicate. An eight-point calibration curve was prepared using three-fold dilutions, starting with a prepared standard sample and two controls with known concentration ranges 0.7–6.9 ng/ml and 7.0–17.0 ng/ml, respectively. The assay sensitivity was 0.37 ng/ml with the intra- and inter-assay precision variations less than 11% and 15%, respectively. The absorption was read using GloMax®-Multi Detection System microplate reader (Promega Corporation, Madison, WI, USA) at 450 nm against 620 nm as a reference.

Plasma follistatin levels were measured by 6-h solid-phase Quantikine ELISA (R&D Systems, Minneapolis, MN, USA) according to the protocol. Samples were analyzed in duplicate. An eight-point calibration curve was prepared using three-fold dilutions, starting with a prepared standard sample of 16.000 pg/ml. The detectable dose ranged from 10 to 83 pg/ml with a mean of 29 pg/ml, and the intra- and inter-assay variations were less than 3% and 10%, respectively. The absorption was read using GloMax®-Multi Detection System microplate reader (Promega Corporation, Madison, WI, USA) at 450 nm against 560 nm as a reference.

Plasma GDF-11 levels were measured by 5-h Human Growth/differentiation factor 11 ELISA kit (Wuhan EIAab Science Co., Ltd., Wuhan, China) according to the protocol. Samples were analyzed in duplicate. An eight-point calibration curve was prepared using three-fold dilutions, starting with a prepared standard sample of 1000 pg/ml. The detectable dose ranged from 15.6–1000 pg/ml with the sensitivity less than 10 pg/ml. The intra- and inter-assay variations were ≤ 4.7% and ≤ 6.9%, respectively. The absorption was read using GloMax®-Multi Detection System microplate reader (Promega Corporation, Madison, WI, USA) at 450 nm.

### Statistical analysis

Statistical analysis was carried out using Statistica 12 software. The data was verified for normality of distribution and equality of variances. Variables that did not meet the assumption of normality were analyzed with nonparametric statistics. The one-way analysis of variance (ANOVA) and Kruskall-Wallis test were used to compare groups. Spearman correlations were used to measure the strength and direction of the relationship between two variables. The limit of significance was set at *p* = 0.05 for all analyses.

## Results

Baseline characteristics of the participants is presented in Table 1. Subjects ranged in age from 61 to 89 years with the mean age of 69 years, who were mostly women (56 females, 45 males). Men were characterized by higher body mass and height than women, however BMI was virtually the same. Women had higher percentage of body fat measured by both methods used in the study than men. Men had wider WC, higher fat free mass and muscle mass than women. Males were also characterized by higher muscle strength and power of upper and lower extremities than women. Men were slightly faster in TUG performance. Women performed better in MMSE, had higher (i.e. worse) GDS scores and experienced higher number of falls per year than men. Women had higher plasma levels of myostatin and GDF11 than men. Men had higher plasma level of follistatin than women.

**Table 1:** Baseline characteristics of the participants (*n* = 101)

	Women	Men
	*n* = 56	*n* = 45
Age (y)	68.2 ± 4.4	70.9 ± 6.8
Body mass (kg)	71.1 ± 12.8	83.7 ± 14***
Body Mass Index (kg/m^2^)	28.3 ± 4.7	28.5 ± 4.0
Waist circumference (cm)	89.5 ± 10.8	101 ± 10.6***
Waist-to-hip ratio	0.86 ± 0.08	0.99 ± 0.06***
Body fat %	43.1 ± 8.1	24.75 ± 4.6***
Fat free mass (kg)	42.6 ± 3.9 (*n* = 52)	60.3 ± 8.9 (*n* = 41)***
Fat free mass (%)	61.1 ± 6.8 (*n* = 52)	73.1 ± 4.7 (*n* = 41)***
Fat mass (kg)	28.3 ± 9.9 (*n* = 52)	22.7 ± 7.3 (*n* = 41)**
Fat mass (%)	38.9 ± 6.8 (*n* = 52)	26.9 ± 4.7 (*n* = 41)***
Muscle mass (kg)	18.1 ± 1.8 (*n* = 52)	29.4 ± 4.1 (*n* = 41)***
ADL (pts)	5.8 ± 0.4	5.9 ± 0.23
IADL (pts)	7.9 ± 0.2	7.93 ± 0.27
Timed Up & Go test (s)	6.2 ± 0.97	6.53 ± 1.99
Tinetti test (pts)	27.6 ± 1.1	27.58 ± 1.7
MMSE (pts)	29.9 ± 0.1	29.4 ± 1.2***
GDS (pts)	2.8 ± 2.6	2.33 ± 2.53
Falls (n/year)	0.6 ± 0.7	0.24 ± 0.5*
Handgrip strength L (kG)	29.5 ± 6.7	45.6 ± 9.97***
Handgrip strength R (kG)	30.5 ± 7.3	47.4 ± 10.8***
Leg Extension 1RM R (kG)	32.3 ± 9.4	55.5 ± 13.4***
Leg Extension 1RM L (kG)	31.2 ± 8.8	54.1 ± 13.5***
Leg Press 1RM (kG)	134.1 ± 30.9	215.8 ± 56.3***
P40% LE R (W)	138.6 ± 41.5	260.7 ± 76.9***
P70% LE R (W)	154.5 ± 47.9	302.1 ± 87.7***
P40% LE L (W)	133.9 ± 40.2	242.8 ± 72.7***
P70% LE L (W)	144.5 ± 44.1	272.1 ± 83.6***
P40% LP (W)	612.4 ± 175.9	1165.8 ± 321.8***
P70% LP (W)	695.7 ± 202	1259.3 ± 338.1***
P_max_ (W)	271.4 ± 88.4	446.2 ± 159.5***
P_max_·kg^−1^ (W·kg^−1^)	3.9 ± 1.2	5.4 ± 1.9***
υ_opt_ (rpm)	71.5 ± 12.6	83.7 ± 18.2***
Myostatin (ng/ml)	42.9 ± 25.6	39.8 ± 17.4
Follistatin (pg/ml)	1429.6 ± 436.9	1695.5 ± 659.6*
GDF11 (pg/ml)	52.5 ± 24.2	40.2 ± 19.4*

Data presented as mean + SD

*Note. ADL* Activities of daily living, *GDF11* growth/differentiation factor 11, *GDS* Geriatric Depression Scale, *IADL* Instrumental Activities of daily living, *MMSE* Mini-Mental State Examination

*p < 0.05, **p < 0.01, ***p < 0.001

Spearman correlation coefficients for females are presented in Table 2. The plasma level of myostatin was negatively correlated with left handgrip strength and υ_opt_. The plasma level of follistatin was negatively correlated with P_max_, P_max_∙kg^− 1^ and P70% leg press, and positively correlated with the TUG test. The plasma level of GDF11 was negatively correlated with body mass, BMI, WC, fat mass and the percentage of body fat measured by both methods. Interestingly, GDF11 correlated positively with the percentage of fat free mass. All the correlations observed for women were at weak or moderate level of strength.

**Table 2:** Spearman correlation coefficients for women (*n* = 56)

	Myostatin (ng/ml)	Follistatin (pg/ml)	GDF11 (pg/ml)
Age (y)	− 0.039	0.185	− 0.089
Body mass (kg)	0.002	− 0.070	− 0.301*
Body Mass Index (kg/m^2^)	0.022	− 0.013	− 0.354**
Waist circumference (cm)	− 0.043	0.083	− 0.372**
Waist-to-hip ratio	−0.103	0.260	−0.202
Body fat %	−0.084	0.035	−0.458***
Fat free mass (kg) (*n* = 52)	−0.075	−0.173	− 0.128
Fat free mass (%) (*n* = 52)	0.073	−0.083	0.359**
Fat mass (kg) (*n* = 52)	−0.071	0.006	−0.331**
Fat mass (%) (*n* = 52)	−0.073	0.083	−0.359**
Muscle mass (kg) (*n* = 52)	−0.025	−0.123	− 0.199
ADL (pts)	−0.024	0.161	0.016
IADL (pts)	−0.132	0.114	0.081
Timed Up&Go test (s)	−0.071	0.366**	−0.163
Tinetti test (pts)	0.163	−0.199	−0.127
MMSE (pts)	0.038	−0.138	0.038
GDS (pts)	0.040	0.034	0.049
Falls (n/year)	−0.239	0.066	−0.133
Handgrip strength L (kG)	−0.296*	−0.063	− 0.080
Handgrip strength R (kG)	−0.203	0.036	0.030
Leg Extension 1RM R (kG)	−0.049	−0.075	− 0.143
Leg Extension 1RM L (kG)	−0.051	− 0.204	−0.170
Leg Press 1RM (kG)	0.031	−0.216	−0.135
P40% LE R (W)	−0.056	−0.179	− 0.124
P70% LE R (W)	−0.169	− 0.135	−0.206
P40% LE L (W)	−0.058	−0.151	− 0.242
P70% LE L (W)	−0.046	− 0.147	−0.151
P40% LP (kG)	−0.091	−0.216	− 0.177
P70% LP (kG)	−0.117	− 0.279*	−0.250
P_max_ (W)	−0.141	−0.387**	− 0.186
P_max_·kg^−1^ (W·kg^−1^)	−0.156	− 0.405**	−0.049
υ_opt_ (rpm)	−0.329*	−0.183	− 0.220
Myostatin (ng/ml)	–	−0.061	0.080
Follistatin (pg/ml)	−0.061	–	0.032
GDF11 (pg/ml)	0.080	0.032	–

*Note. ADL* Activities of daily living, *GDF11* growth/differentiation factor 11, *GDS* Geriatric Depression Scale, *IADL* Instrumental Activities of daily living, *MMSE* Mini-Mental State Examination

*p < 0.05, **p < 0.01, ***p < 0.001

Spearman correlations coefficients for males are presented in Table 3. There were no correlations observed between plasma levels of myostatin, follistatin, GDF11 and measured parameters. The similar trends as in females were observed in men, but the correlations were too weak to reach the statistically significant level.

**Table 3:** Spearman correlation coefficients for men (*n* = 45)

	Myostatin (ng/ml)	Follistatin (pg/ml)	GDF11 (pg/ml)
Age (y)	0.007	−0.021	−0.159
Body mass (kg)	−0.009	−0.058	− 0.104
Body Mass Index (kg/m^2^)	−0.050	− 0.075	−0.164
Waist circumference (cm)	0.029	0.009	−0.131
Waist-to-hip ratio	−0.028	0.143	−0.000
Body fat %	−0.074	−0.119	− 0.036
Fat free mass (kg) (*n* = 41)	−0.174	0.041	0.028
Fat free mass (%) (*n* = 41)	−0.162	−0.023	0.078
Fat mass (kg) (*n* = 41)	0.052	0.020	−0.020
Fat mass (%) (*n* = 41)	0.162	0.023	−0.078
Muscle mass (kg) (*n* = 41)	−0.119	0.037	0.029
ADL (pts)	0.216	0.040	0.057
IADL (pts)	−0.181	−0.227	−0.233
Timed Up&Go test (s)	−0.101	0.139	−0.031
Tinetti test (pts)	−0.046	0.092	−0.254
MMSE (pts)	−0.017	0.114	0.293
GDS (pts)	0.209	0.154	0.117
Falls (n/year)	0.091	0.171	0.131
Handgrip strength L (kG)	0.153	−0.202	0.044
Handgrip strength R (kG)	0.113	−0.110	−0.015
Leg Extension 1RM R (kG)	−0.062	−0.264	− 0.045
Leg Extension 1RM L (kG)	−0.187	− 0.193	−0.135
Leg Press 1RM (kG)	0.034	−0.052	−0.051
P40% LE R (W)	−0.016	−0.251	0.055
P70% LE R (W)	0.008	−0.230	0.101
P40% LE L (W)	−0.196	−0.232	− 0.132
P70% LE L (W)	−0.189	− 0.253	−0.128
P40% LP (kG)	−0.022	−0.178	0.163
P70% LP (kG)	−0.008	−0.085	− 0.04
P_max_ (W)	−0.101	− 0.127	−0.013
P_max_·kg^−1^ (W·kg^−1^)	− 0.156	−0.115	− 0.017
υ_opt_ (rpm)	−0.094	0.016	−0.127
Myostatin (ng/ml)	–	0.008	0.183
Follistatin (pg/ml)	0.008	–	0.171
GDF11 (pg/ml)	0.183	0.171	–

*Note. ADL* Activities of daily living, *GDF11* growth/differentiation factor 11, *GDS* Geriatric Depression Scale, *IADL* Instrumental Activities of daily living, *MMSE* Mini-Mental State Examination

*p < 0.05, **p < 0.01, ***p < 0.001

## Discussion

This is the first study which investigates whether the circulating plasma proteins: myostatin, follistatin and GDF11 are related to muscle strength, power and υ_opt_ in older adults. We demonstrated the inverse relationship between myostatin and muscle strength of the upper extremities and between myostatin and quadriceps υ_opt_ in women but not in men. We found the negative correlation between follistatin and muscle power in older women. We also demonstrated a distinctive link between GDF11 and body composition in women but not in men.

Myostatin is known as a key negative regulator of muscle mass. Loss of function of myostatin induces skeletal muscle hyperplasia and hypertrophy [5]. Since its discovery, it became a unique and desirable therapeutic target. It can be interfered by neutralizing its activity antibodies [5]. Becker et al. [28] reported the increase of lean body mass and improvement of some performance measures in older individuals after the treatment by the humanized monoclonal myostatin antibody LY2495655. In future, it may be potentially indicated for treatment of hip arthroplasty, cancer cachexia, and elderly fallers [29]. Serum myostatin has been reported to increase, decrease or remain unchanged with age [1, 30–33]. Bowser et al. [34] showed in mice that there is age-associated increase in myostatin levels and the myostatin:follistatin ratio in slow-twitch soleus muscle and reversed pattern in the fast-twitch extensor digitorum longus muscle. Conflicting results have also been provided considering association of myostatin to body composition, muscle mass and strength as well as physical performance [2, 30, 31, 35]. Binns et al. showed that neither serum myostatin nor protein intake influenced the total body lean mass among older men and women [36]. Bergen et al. [33] obtained a significant positive correlation with grip strength and knee extensor strength in young men but not in older men or women. However, Han et al. [32] reported the negative correlation between handgrip strength and serum myostatin level in hemodialysis patients. The accelerator-brake (or yin and yang) hypothesis has been put forward to explain on one hand, the restrictive myostatin activity to excessive muscle growth (role of chalone) and on the other hand, lower myostatin expression in response to unfavorable metabolic environment, e.g. metabolic syndrome, inflammatory cytokines or uremia [32, 35, 37–39]. Undoubtedly, the potential modulating role of androgens and estrogens on myostatin and other proteins that maintain muscle function is of interest. Testosterone is one of the well-known anabolic hormones, which can increase muscle protein muscle synthesis and muscle mass [40]. Lakshman et al. [41] showed significant correlation between free testosterone and myostatin levels in younger men. On the contrary, Smith et al. [40] found neither testosterone nor estradiol have any effect on myostatin mRNA expression in postmenopausal women. According to the other study, the increased level of estradiol correlates with decreased level of myostatin mRNA expression in younger females [42]. Further studies are necessary to interpret those conflicting reports on the role of androgens and estrogens in the activity of myostatin and other muscle-related proteins.

Another factor should be mentioned, such as activin A, which is thought to replicate a biological activity of myostatin on skeletal muscle. Gilson et al. [43] demonstrated that follistatin-induced muscle hypertrophy resulted in activin A inhibition in wild-type mice. In addition, it was shown that human anti-ActRII antibody bimagrumab (BYM338) inhibits myostatin- and activin A- muscle atrophy and significantly increases skeletal muscle mass in mice [44]. Activin A and myostatin bind to type II activin receptors with greater affinity of activin A to the type IIA. Activin A circulates in the bloodstream and its concentration increases in acute conditions such as inflammation, respiratory and renal failure, and some types of cancer [34, 45]. Baccarelli et al. [46] showed that increased serum concentration of activin A is associated with age in both men and postmenopausal women. Activin A was not a subject of this study; however, it might be useful to consider examining it in future analyses.

Follistatin was demonstrated to prevent myostatin binding to the activin type II B receptor, which as a result neutralizes its activity in circulation. It was observed that follistatin overexpression in mice promotes increase in muscle mass [7]. Similarly to myostatin, in the majority of recent studies, follistatin was not found to be age-dependent [2, 31], though this data is also conflicting [33]. In several studies follistatin was not found to reflect dynapenia in older women or men [2, 31]. Interestingly, Miyamoto et al. reported negative association between plasma follistatin and muscle strength in patients with chronic kidney disease, which is not consistent with observed effects of follistatin in inducing muscle hypertrophy [38]. Likewise, Liaw et al. [35] found a negative correlation between follistatin and gait speed among older adults. On the other hand, follistatin increased after resistance training in older women and performance gains have been attributed to the blocked degradation pathways via follistatin [47]. All these discrepancies may be explained by the fact that increased follistatin may accompany elevated myostatin levels [38]. Increased follistatin levels occur also in inflammatory diseases and have been suggested to counteract catabolism in chronic kidney disease [35, 38].

GDF11 and myostatin are closely related TGF-β superfamily proteins [48]. Their homology is very impressive, differing by 11 residues within the amino acid sequence. Nevertheless, myostatin is expressed primarily in skeletal muscle and acts to limit muscle growth. GDF11 is expressed more widely and plays multiple roles [49], including suggested aging regulation of multiple mammalian tissues [48]. Unlike myostatin, GDF11 declines in aging mice [50]. In contrast, Schafer et al. [1] showed that GDF11 levels do not decline throughout aging and there was no difference between sexes in healthy adults.

GDF11 has been proposed to be both a rejuvenating factor and a biomarker of advanced biological aging [1, 3]. Using parabiosis (exposure of the aged mouse to a young circulation), Sinha et al. [50] demonstrated that circulating GDF11 is a rejuvenating factor for skeletal muscle. Parabiosis (increased GDF11 levels in aged mice) reversed functional impairments and restored genomic integrity in aged muscle stem cells and increased strength and endurance exercise capacity [50]. The 4-week exposure to the blood circulation of young mice resulted in the cardiac hypertrophy regression in old mice. GDF11 was identified as a responsible circulating factor. Restoration of GDF11 in old mice to youthful levels repeated the effects of parabiosis and reversed age-related hypertrophy [51].

Nevertheless, there are considerable new data showing that GDF11 can aggravate rather than regenerate skeletal muscle injury in old animals [3]. It was shown to inhibit muscle regeneration in a dose-dependent manner [52]. There are also doubts whether GDF11 therapy can reverse cardiac pathologies while elevated blood levels of GDF11 may generate a cachectic effect in skeletal and cardiac muscles in both young and old animals [3]. In one clinical study, Schafer et al. [1] demonstrated that older adults with slow gait, weak grip strength and higher prevalence of cardiac conditions had higher GDF11 levels. In the same study individuals with higher body weight were characterized by a trend towards lower GDF11 plasma levels. These authors suggested that GDF11 circulating levels may be associated with deficits in multiple physiological functions [1].

Age-related gradual decline of muscle loss associates more with fast twitch (type II) fibers than with type I ones. Fast twitch fibers mainly determine velocity of contraction and muscle power [27]. Therefore, these two measures have been proposed to be more crucial factors determining functional performance than muscle strength [16, 17]. To the best of our knowledge our study is the first one to report on the relationship of directly measured muscle power and optimal shortening velocity to circulating myostatin, follistatin and GDF11. In one study with young non-athletic men, the polymorphism in the myostatin gene was associated with the ability to produce peak power assessed with vertical jumps [53]. In a large clinical trial, Becker et al. [28] reported the improvement of power-demanding performance measures (stair climbing, five-chair rise, fast gait speed) in the older individuals after the treatment with the humanized monoclonal antibody LY2495655. For less power-intensive performance-based measures (6-min walking distance, usual gait speed) and muscle strength no important treatment effects were observed [28].

Our data show a consistently negative trend for the association of all the three proteins to muscle function measures, with significant relationship of myostatin to υ_opt_ and of follistatin to P_max_, P_max_∙kg^− 1^ and P70% leg press in women. For muscle strength, only one significant relationship was found for the myostatin-left handgrip strength association in women, with no significant relationship of circulating proteins to lower extremity isometric strength measures. Therefore, power and shortening velocity might be more affected by circulating myostatin and follistatin than isometric strength. This association being also more visible in older women than in men.

In our study GDF11 was inversely related to body mass, BMI and the percentage of body fat in women. Positive relationship to the percentage of fat free mass may be related to the strong negative association between the percentage of fat free mass and body mass in women (*r* = − 0.86; *p* < 0.0001). Therefore, lower body mass was related to higher relative percentage of fat free mass. The association between GDF11, absolute values of fat free mass and muscle mass tended to be negative. This supports the data of some previous animal and clinical studies [1, 3] and suggests that GDF11 circulating levels may be associated with increased catabolism in older adults. Only a minor relationship to functional tests (the only significant relationship being between follistatin and TUG test in women) may be related to the fact that our subjects were highly functioning elders with no apparent functional limitations.

Sex-related differences observed in this study are in accord with some previous reports. Schirwis et al. [54] reported that the effects of myostatin deficiency on maximal force and power are greater in young (as compared to old) and female (as compared to male) mice. Bergen et al. [33] showed that older females compared to younger have higher myostatin levels and older men have lower myostatin levels compared to younger ones. Nevertheless, one should note that our data show a consistently negative trend for the association of all the three proteins to muscle function measures both in women and men. Future epidemiological studies with larger samples of participants should solve this problem and elucidate whether these associations are valid in both sexes.

The main strength of this study was the fact that it considered simultaneously the relationship between plasma circulating proteins myostatin, follistatin, and GDF11 and muscle strength and power in older adults. Several limitations of our study should be considered. One limitation was the relatively small number of the participants. Another one is the cross-sectional nature of analysis performed in this study and modest correlations presented as uncorrected for multiple comparisons. Participants were volunteers, usually more healthy and willing to participate than general population of older subjects. Possible coexisting factors such as inflammation should also be considered in the design of future prospective studies. Finally, we used commercially available GDF11 reagent and ongoing discussion concerning the validity of different reagents should be acknowledged.

## Conclusions

The circulating myostatin and follistatin are negatively associated with muscle function in older women. The relationship between circulating plasma proteins is more visible for muscle power than muscle strength. GDF11 appeared to have a higher association with the body mass and composition than muscle function in older women. Future studies should explore whether these changes are the adaptation to age-related increased catabolism and whether they are potentially reversible.

## Abbreviations

1RM: one repetition maximum
ADL: Activities of Daily Living
BMI: body mass index
CC: calf circumference
F: force
GDF11: growth/differentiation factor 11
GDS: Geriatric Depression Scale
HC: hips circumference
IADL: Instrumental Activities of Daily Living
MMSE: Mini-Mental State Examination
P: power
P_max_: maximum power
rpm: rotations per minute
TGFβ: transforming growth factor-β
TUG: Timed Up & Go
WC: waist circumference
υ: velocity
υ_opt_: optimal shortening velocity
